# Service-learning’s impact on dental students’ attitude to community service

**DOI:** 10.1186/s12909-023-04045-2

**Published:** 2023-01-25

**Authors:** Hammam Ahmed Bahammam, Sarah Ahmed Bahammam

**Affiliations:** 1 Department of Pediatric Dentistry, Faculty of Dentistry, King Abdulaziz University, P.O. Box 80209, Jeddah, Saudi Arabia; 2 Department of Pediatric Dentistry and Orthodontics, College of Dentistry, Taibah University, Universities Road, P. O. Box, 344 Medina, Kingdom of Saudi Arabia

**Keywords:** Service-learning, Dental students, Community service, Attitude, Impact

## Abstract

**Background:**

This study aimed to observe the impact of service learning on the attitude of senior dental students toward community service.

**Methods:**

A cross-sectional survey-based was conducted and recruited a total of 120 senior students of Dentistry Taibah University that were enrolled in a clinical service-learning course using convenience sampling. The attitude of dental college students toward community service based on the model of helping behavior by Schwartz was evaluated through Community Service Attitude Scale (CSAS). The data was collected using the same questionnaires for the pre-test and post-test. Data were analyzed by using SPSS 25. A repeated-measures mixed-model ANOVA was used to test the changes across pre-and post-test.

**Results:**

A total of 96 students completed the first survey, making a response rate of 80%, and 78 among them completed the second survey as well, making a response rate of 81%. Significant change (*P* < 0.0001) in ANOVA indicated that there was an overall change in attitude.

**Conclusions:**

It can be concluded that community-based service-learning positively impacts the attitude of last year’s dental students toward understanding needs at the community level and the attitude to help in providing dental care at the community level.

## Background

Oral health is important for overall health, but not everyone has access to the oral health care services they require [1]. Vulnerable populations in society including very young children, the elderly, and poor people with diagnosed medical diseases, are at risk of poor oral hygiene because they face barriers to oral health care [2]. This oral health discrimination can be alleviated by trained dental healthcare professionals who are willing to treat it [3]. Dentists’ willingness to treat and assist the underprivileged can be wrought during their studies [4]. Alraqiq et al. [5] postulated that altruism in dental students is highly correlated with their exposure to the less privileged population outside the clinical setting.

Many students are changing their learning objectives and goals. While it is expected of students to grasp the fundamentals, there is an increasing demand for students to apply what they have learned to challenges in the outside world. They must be knowledgeable about topics outside of the conventional academic fields. Additionally, to integrate into the diverse community and make the move from college to the workplace smoothly and easily, they must cultivate their critical thinking abilities and teamwork abilities [6]. With the passage of time, the learning content changes, and so must the teaching methodologies and learning. Community-based learning, also known as service learning, is one such learning and teaching methodology that meets the above criteria [7]. It is the type of teaching that combines instruction with meaningful community service opportunities. Service learning is a planned learning activity or community-centered education that is recommended for achieving academic goals while also learning community service. Reflection, community partner participation, and maintaining a balance between learning and service are all important components of service learning [8, 9]. It exemplifies an approach that connects young people with institutions that serve the larger community. Furthermore, it can provide them with the opportunity to apply newly acquired skills and knowledge in real-life situations or communities. Furthermore, community-based learning extends what students learn outside of the classroom and into the community, assisting them in developing a sense of caring for others [10]. John Dewey emphasized the importance of reflection and developed the concept of service-learning and strongly believed in developing a sense of commitment and ability to positively contribute to society [11].

Schwartz’s model is used as the theoretical framework to examine the relationship between students’ attitudes and behavior to help in their service learning. There are four steps in this model [12, 13]. The first step is activation. In this step, the students or volunteers understand the needs of the people and they respond by being aware of their needs and feeling connected to them. The second step is the obligation step, where there the students feel a moral obligation to respond to the people’s needs with empathy while maintaining personal and situational norms. The third step is defense, where the pros and cons of helping others are reconsidered and the situation is reassessed to have a serious response to people’s needs. The last fourth step is a response, where the intention to do community service is developed.

In line with this, service-learning has been used at dentistry schools for more than a decade to support dental students’ professional development and to help them ‘integrate’ their responsibilities to the community as healthcare providers by combining educational objectives and community engagement [14, 15]. The impact of dental education on student attitudes toward underprivileged populations, according to Coe et al., has demonstrated that [16] senior dental students’ positive opinions toward a homeless population improved further after performing seven weeks of community service. Studies on the impact of education generally and community-based education specifically on medical students’ attitudes toward marginalized people have shown that some students and residents experience a decline in views and idealism as they advance in their training [16].

According to a recent study on dental students’ perspectives on community-based service conducted in Iran [17], the students felt it aided in their professional and personal development, as well as broadened their perspective and responsibilities toward preventative dentistry. According to Ebrahim and Julie [18], although self-reflection had a significant influence on students’ attitudes toward service and community, there was no increase in dentistry students’ caring attitudes for the underprivileged based on community service after participating in the program.

Previous research has emphasized the importance of improving dental students’ attitudes toward assisting the underprivileged by employing service learning as an instructional strategy. Few studies, however, show a clear link between service learning as an educational approach and its impact on dental students’ attitudes toward community service [19–21]. Therefore, the current study was conducted to evaluate the impact of service learning on the attitude of senior dental students toward community service.

## Methods

A cross-sectional survey-based design was used for this study. Ethical clearance was obtained from Research Ethical Committee at the College of Dentistry Taibah University and informed consent was taken from the participants. A total of 120 senior students of Dentistry Taibah University that were enrolled in a clinical service-learning course were approached for the survey. It is mandatory specifically for senior dental students to spend an average of 25 days on a rotation basis at 12 different external sights assigned by the university. Students’ s service learning is evaluated and strengthened through self-reflection, including blog posts, journal entries, and self-evaluation. The course modules were similar as reported by Brondani et al. [14] with their primary objectives. The length and learning activities in each module are different.

Convenience sampling was used for the enrolment They were provided information about the aims and objectives of the study and were given two survey forms to which they had the autonomy to respond or decline. The attitude of dental college students toward community service based on the model of helping behavior by Schwartz was evaluated based on Community Service Attitude Scale (CSAS) [22].

The Community Service Attitudes Scale (CSAS) was developed in stages, according to a study. Schwartz’s model of altruistic helpful behavior served as the foundation for the CSAS items [13, 23, 24]. Altruistic helpful conduct reflects how attentive people are to the needs of others and how eager they are to assist others. The concept is made up of cognitive and affective processes that a person takes to advance, starting with the awareness of a need and concluding with an overt offer of assistance. Each phase influences the subsequent one, thus the person advances to Phase 2 if Steps 1 through 4 of Phase 1 have all been triggered. Phase 2 leads directly into Phase 3. Phase 4 is the final stage, where the choice to perform community service is decided. As a result, questions measuring each phase of the Schwartz model’s community service attitude were developed in this manner [13].

The data for this study was collected using the same questionnaires for the pre-test and post-test from March 2021 to May 2021. The first survey was conducted immediately after completing the service learning program. The retrospective pre-test (RPT) was filled out by the respondents of post-survey after three weeks of completion of the program to avoid response shift bias. Overall Cronbach Alpha for CSAS Score Construct is 0.765. Whereas, for the pre-test, the questionnaire was to be filled out retrospectively to report on their attitude before taking the program.

The attitude toward community service in 8 scales (i.e., normative helping behavior, connectedness, awareness, benefits, career benefits, costs, seriousness, and intention to community service) was assessed based on 46 items in the survey questionnaire. The study tool adopted in this study was similar to the instrument used by Shiarella et al. [22] and Coe et al. [16]. Shiarella et al. created questionnaire questions that corresponded to the Schwartz model’s sequential phases, tested them and ran a factor analysis on them to create the eight CSAS scales, which total 46 items. The questionnaire comprised student demographics that included; gender, age, enrolment in the International Dentist Program, and involvement in volunteer activities. Other questions assessing the attitude of senior dental students toward community service were based on a 7-point Likert scale; 7 = extremely likely, 6 = quite likely, 5 = slightly likely, 4 = neither likely nor unlikely, 3 = slightly unlikely, 2 = quite unlikely, and 1 = extremely unlikely. The questionnaire included questions assessing eight CSAS scores in the same sequence as Shiarella et al. instrument. Questions 1–12 used seven-point anchor scales of extremely likely: being 7, quite likely, slightly likely, neither likely nor unlikely, slightly unlikely, quite unlikely, and extremely unlikely: being 1, and questions 13–46 used seven-point anchor scales of strongly agree: being 7, agree, slightly agree, neither agree nor disagree, slightly disagree, disagree, and strongly disagree: being 1.

The data was entered and analyzed using Statistical Package of Social Sciences (SPSS) version 25.0. Descriptive statistics were calculated for each study variable. The pre-and post-test attitude scores were presented as outcome variables for all eight scales. A repeated-measures mixed-model ANOVA was used to test the changes across pre-and post-test, which highlighted the dependencies between the eight scales across the two occasions and was applied on matched post- and pre-test. The level of significance was considered to be < 0.05.

## Results

A total of 96 students completed the first survey, making a response rate of 80%, and 78 among them completed the second survey as well, making a response rate of 81%. Table 1 demonstrates the descriptive characteristics of the respondents who filled post-test survey questionnaire. Out of 96 respondents, 42 (43.7%) were female and the majority 54 (56.3%) respondents were male. The age ranged from 25 to 36 with an average of 26.5 years. The gender and age distribution of the respondents was similar to the 120 entire class students that were invited to participate (*P* > 0.05). The average age for the whole class was 27.1 years, among them 42.5% were female and 58.5% were males. Similarly, statistically, there was no significant difference (*P* > 0.26) in age or gender in the participants that filled out pre and post-surveys (*n* = 78) and those that only filled out the post-test survey (*n* = 18). Among post-test respondents, 46.9% (*n* = 45) reported having a prior experience of regularly being engaged with volunteer work or community work. Whereas, only 29.1% (*n* = 28) reported being regularly engaged with extracurricular volunteer/community work along with dental school. This indicates that there was a decrease in regular volunteers during dental school when compared to before taking admission to a dental course.

**Table 1:** Demographics of post-test respondents (*n* = 96)

Characteristics		Post-test respondents (%)
Gender		
	Male	54 (56.3)
	Female	42 (43.7)
Age		
	≤25	15 (15.6)
	26-29	58 (60.4)
	≥30	23 (24)
Prior Community/Volunteer work		
	Never	14 (14.6)
	Occasionally	37 (38.5)
	Regularly	45 (46.9)
Community/Volunteer work with dental school		
	Never	21 (21.9)
	Occasionally	47 (48.9)
	Regularly	28 (29.2)

**Fig. 1:**
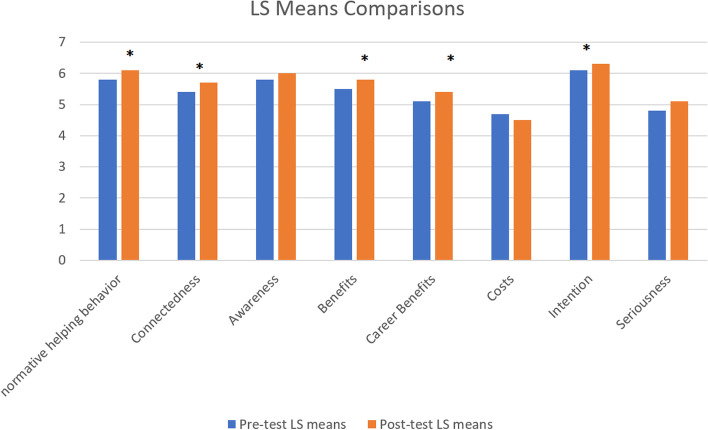
Pre-test and post-test change. (a) *denotes change in post-test and pre-test with P-value < 0.05 (b) For scales of career benefits, costs, and benefits, the vertical axis numbers correspond to anchor scales as follows. 1: Extremely unlikely, 2: Quite unlikely, 3: Slightly unlikely, 4: Neither likely nor unlikely, 5: Slightly likely, 6: Quite likely, 7: Extremely likely. (c) For all other scales, the vertical axis numbers correspond to anchor scales as follows. 1: Strongly disagree, 2: Disagree, 3: Slightly disagree, 4: Neither agree nor disagree, 3: Slightly agree, 2: Agree, 1: Strongly Agree

Table 2 represents the change impact (post-test and pre-test) of service learning on the attitude of students. After applying the repeated measures mixed model ANOVA the overall F was found significant (*P* < 0.001). It summarizes the change score for each occasion based on least squared (LS) means calculated by repeated measures mixed model ANOVA using 56 responses (matched post- and pre-test). Overall, the change was observed on the two occasions when all variables were considered simultaneously (*P* < 0.0001). Whereas, the *P*-values for the eight scales can be seen individually in Table 2. Here, normative helping behavior had a pre-test mean of 5.823 which increased in the post-test with a mean of 6.125 having a significant difference of 0.302 (*P* = 0.0021). The other significant changes were seen in connectedness, benefits, career benefits, and intention. However, a significant difference was not observed in awareness, costs, and seriousness. Similarly, these findings can be seen visually in Fig. 1. The time*scale interaction was used to test the possibility of change in selected scales across time. Here, the time*scale interaction had a *p*-value of 0.058 which indicates that there was not adequate confirmation that there was a dissimilar measure of change across the scale, i.e., the scale did not impact the service-learning program. Service learning has changed all the scales.

**Table 2:** Pre-test and Post-test change

Occasion		LS Mean	SE	95% Confidence Interval		*P*- value
Normative helping behavior	Pre-test	5.82 ± 1.20	0.08	5.430	5.634	0.002*
	Post-test	6.12 ± 1.18	0.07	5.722	6.343	
Connectedness	Pre-test	5.41 ± 0.52	0.12	5.050	5.775	0.001*
	Post-test	5.69 ± 1.54	0.11	5.375	6.133	
Awareness	Pre-test	5.83 ± 0.91	0.09	5.543	6.235	0.06
	Post-test	5.98 ± 0.65	0.08	6.000	6.123	
Benefits	Pre-test	5.57 ± 0.85	0.12	5.455	6.000	0.002*
	Post-test	5.82 ± 1.18	0.11	5.654	6.175	
Career benefits	Pre-test	5.12 ± 1.01	0.13	4.881	5.157	0.004*
	Post-test	5.37 ± 1.91	0.11	5.234	5.578	
Costs	Pre-test	4.68 ± 1.12	0.14	4.190	4.690	0.23
	Post-test	4.51 ± 0.95	0.13	4.150	4.450	
Intention	Pre-test	6.14 ± 0.51	0.10	5.750	6.150	0.04*
	Post-test	6.35 ± 1.25	0.09	5.810	6.160	
Seriousness	Pre-test	4.84 ± 0.69	0.13	4.550	5.110	0.33
	Post-test	5.18 ± 0.75	0.12	4.500	5.425

## Discussion

The purpose of this study was to determine the effect of service learning on senior dental students’ attitudes toward community service. There have been very few studies that examined the impact of service-learning programs on senior dental students’ attitudes toward community services using a validated scale. Few studies have found that service learning has a positive impact on college students’ attitudes toward social responsibility [25]. Hatton and Mandrusiak [26] discovered a positive impact of service learning on physiotherapy students in the care of the elderly. Shiarella et al. [22] validated this questionnaire and scale, and Coe et al. [16] successfully used it on dental students.

In the current study, a significant change in repeated measure mixed model ANOVA between the pre-and post-test was seen indicating that there was an overall change in attitude. Also, after adjustment for the scales effect, the LS mean for five scales (i.e., normative helping behavior, correctness, benefits, career benefits, and intention) improved and showed a significant level of change. Whereas, after community service, the overall attitude of the dental students at the individual level changed and positively impacted their attitude. However, this impact was not dependent on the scale and it was found non-significant.

In a study conducted in India [27], 99% of participants agreed that they must assist the community, and 97% of dental students felt that the outreach experience had made them aware of their obligations in the community. Similar findings were seen in two more studies, where 94% [28] and 86% [29] of participants concurred that the outreach experience had increased their awareness of their social responsibility.

In our study, with a LS mean change ranging from 0.338 to 0.153, there was improvement seen in all eight scales. As the pre-test scores were very high, there was limited possibility of improvement in post-scores, as high pre-scores might have given the appearance of a small change due to the ceiling effect. The results of this study are ‘modest’ similar to the results seen in a previous study [21]. However, the findings were in contrast to the study by Holtzman and Seirawan [30] on first-year dental students which found no significant change in the attitude of students due to community service. This might be because first-year dental students had less exposure, which could have led to idealistic attitudes that later shifted to practical ones, resulting in lower attitude scores.

Ferrari and Chapman [31] posit that service learning is different from internship and volunteerism as it is more focused on the enhancement of students’ understanding of their textbook curriculum through community service experience and later on reflection on that gained experience. Whereas, in internships, the beneficiaries are the service providers and the focus is on acquiring career-oriented skills only [32]. Similarly, volunteerism differs from service learning as the main beneficiaries are the recipients and it is more focused on the service concept [33]. In this regard, a study was conducted and revealed that students who were actively engaged in voluntary activities had a high attitude toward community services [34]. This may be because students with greater volunteer experience have more positive opinions about the need for community service and may be more sensitive to the needs of less privileged patients [1]. This was not analyzed in our study and hence it remains one of the limitations of our study.

This study used the approach of retrospectively filling out a form for a pre-test. The retrospective pre-test (RPT) is advised particularly when the evaluation’s objective is to gauge students’ perceptions of change and encourage reflection on personal development as related to the program and is in concordance with the objective of our study. Because the program of interest itself does not affect survey respondents’ internal standards, the RPT is also known to lessen response-shift bias, a typical confounder of the conventional pre and post-test study [35]. When the pre-test result is not available, the RPT can be utilized to establish a baseline [36]. Although overestimation of the impact of the program and recall bias are the potentially associated criticism with this technique, even then it is utilized by the authors as reported in the literature [37–39].

Although service learning is becoming more and more popular, research to date suggests that various obstacles must be solved to quantify the benefits that students receive from engaging in service-learning programs. The first is that such courses tend to be brief [40]. It is challenging to offer a rich experience that can change students’ attitudes when exposure to service learning is restricted. Moreover, surveys of students’ moral development revealed that, although moral development scores did not change, students who participated in service learning courses self-reported that they improved their outlook on life, felt more empathetic, had a better grasp of how to address social issues, and had a stronger desire to make the world a better place [41, 42]. The literature highlights the requirement for a more rigorous research design, which should include, among other things, pre-and post-tests, control groups, and the use of multi-item scales [43]. It is crucial to stress the value of students’ interest in the caliber of service learning activities as well as their greater understanding of the function that service learning plays. Charity, projects, and social transformation were suggested as paradigms by K. Morton [44] as agents that might ultimately transform people and communities.

There were some limitations to the study. Due to the small sample size, multiple comparisons were not performed with statistical confidence, and data were analyzed only for individual items. Also, it was a single-center study, at one particular dental school. The different impacts could have been seen on students enrolled in different learning programs. Future research on various community-based service-learning initiatives and their influence on dentistry students’ attitudes is advised. To improve learning outcomes and a sense of commitment for the underserved in the community, it will be helpful to identify the best program or practice. Future research can be done to follow up on these students as working dentists to comprehend the effects of these programs on the ground.

## Conclusion

As measured with CSAS, senior students in their final year, experienced a change in their attitude after being enrolled and completing a community-based service-learning program. The most significant pre-and post-test change was seen in the scales of normative helping behavior, connectedness, career benefits, intention, and benefits. The results of this study can be used by educators or higher institutes, especially of healthcare origin as an example of the potential beneficial outcome of the community-based service-learning program. Also, the CSAS tool can be used by these institutions to assess the impact of their service-learning program on the attitude of their students.

## Data Availability

The datasets used and/or analyzed during the current study are available from the corresponding author upon reasonable request.

## Abbreviations

ANOVA: Analysis of variance
CSAS: Community Service Attitude Scale.
RPT: Retrospective pre-test.
